# Improving volumetric productivity of a stable human CAP cell line by bioprocess optimization

**DOI:** 10.1186/1753-6561-5-S8-P66

**Published:** 2011-11-22

**Authors:** Ruth Essers, Helmut Kewes, Gudrun Schiedner

**Affiliations:** 1 CEVEC Pharmaceuticals GmbH, Gottfried-Hagen-Str. 62, D-51105 Köln, Germany

## Background

For the production of recombinant proteins, a human cell-derived expression technology can offer significant advantages with respect to protein quality, serum half-life and safety. High volumetric productivity with first-class quality is the ultimate ambition during process development.

CEVEC’s proprietary expression system based on human amniocytes offers significant advantages for the production of complex human proteins and antibodies. The key benefits are the stable and high expression of recombinant proteins with human type posttranslational modifications, the robust growth behaviour with competitive high cell densities and the easy handling in serum free suspension. CAP cells meet all regulatory requirements, they are of non-tumour origin and from an ethically accepted source.

In order to test the performance of CAP cells for the production of very complex proteins, stable C1-inhibitor expressing CAP cells were developed. C1-inhibitor is a serine protease inhibitor (serpin) and one of the most heavily glycosylated plasma proteins bearing numerous complex N- and O-glycans.

## Material and methods

Cells: CAP cells stably expressing C1-inhibitor

Base medium: Protein Expression medium PEM (Gibco #12661013) containing 4mmol*L^-1^ glutamine (Invitrogen #25030024)

Supplements: Soy Peptone E110 (OrganoTechnie #AI885), Glucose (Sigma #G8679), Valproic acid VPA (Sigma #4543)

ELISA: In-house C1-inhibitor ELISA using serum derived C1-inhibitor as standard

SDS-PAGE/Western Blot: In-house Western Blot for C1-inhibitor

To investigate the influence of different hydrolysates and supplements under controlled conditions we use DASGIP´s parallel bioreactor system for cell culture. See table 1 for standard physical process parameters.

**Table 1:** Standard physical process parameters for DASGIP fermentation

process parameter	
T	37°C
V	0.6L
stirring	marine impeller, 120rpm
aeration	sparging, 0.03vvm
pH	7.0 (CO_2_/ 0.5M NaOH)
DO	40% (air saturation)
starting cell density	3-5E5mL^-1^

## Results

Parental CAP cells were transfected with a plasmid containing expression cassettes for the human C1-inhibitor driven by the CMV promoter, and a Blasticidine resistance gene for selection. Out of three stable pools, one pool was selected for further single cell cloning by limiting dilution. From initial 251 single cell clones, the best 5 were chosen for further scale-up. Subsequent process development was carried out with one clone showing the best performance in growth and expression.

First we tested the influence of different media supplements shown before to improve cell growth and productivity in CAP cells. Either additional glucose, glutamine, pyruvate, soy peptone, tryptone plus, tryptone or an in-house mixture of R3-IGF, transferrin, SyntheChol and progesterone were added to the base medium. Cells were cultivated in shake flasks and cell densities, viabilities, metabolites and product concentration were monitored continuously.

Whereas the in-house mixture lead to higher growth rates, the product yields increased upon the addition of glucose (1.2-fold), soy peptone or tryptone (1.5-fold).

Based on these results, we tested additional hydrolysates from cotton seed, wheat gluten, rice protein and soy. Hydrolysates and extra glucose were added to the initial medium and cells were cultivated in shake flasks.

Only medium supplemented with soy peptone II (up to 1.7-fold) and with cotton seed hydrolysate (up to 2.4-fold) showed better performance as compared to the control with extra glucose but without hydrolysate.

In addition to medium supplementation we determined the optimal pH value for good growth rates, high product quantity and quality. The different batches (pH unregulated, pH 7.0, 7.2 or 7.4) did not differ in maximal product concentrations but in product qualities. This was monitored with SDS-PAGE and western blot. Due to additional specific bands at pH7.2 and pH7.4 either caused by degradation or incomplete glycosylation we decided to continue with pH7.0 for following process development steps.

Under controlled conditions additional glucose alone or either with soy peptone I, soy peptone II or cotton seed hydrolysate were supplemented to the base medium. Parallel fermentations with different enriched media were started at pH7.0 and cell densities of 3.0*10^5^mL^-1^ under controlled conditions. Cotton seed hydrolysate increased growth, but highest cell density could be observed with soy peptone II or without hydrolysate addition. The maximum product concentration was higher compared to the control in all peptone-fed fermentations. Supplementing soy peptone II or cotton seed hydrolysate almost doubled maximum product concentration.

As a next step, we examined the effect of valproic acid (VPA) on productivity. VPA is a short-chain fatty acid used as well established drug and classified histone deacetylase inhibitor. We set up two cultures with glucose and soy peptone II and two cultures with glucose and cotton seed hydrolysate. In each case, one of the duplicate cultures was additionally supplemented with 4mmol*L^-1^ VPA.

Initial cell densities were 3.5*10^5^mL^-1^ in all four parallel controlled cultures. The addition of VPA was carried out at viable cell densities of 3.0*10^6^mL^-1^. Addition of VPA resulted in 1.6-2.0-fold increase in productivity compared to the corresponding control cultures without VPA

To determine the product quality we performed Western blots with samples from the supernatant of the batches supplemented with VPA. The highest protein quality was obtained from the Soy peptone II-supplemented cultures.

We confirmed the results with 8 (parallel) runs and observed consistent results for cell densities, growth behaviour, product titers, cell specific and volumetric productivities.

## Conclusions

**Figure 1:**
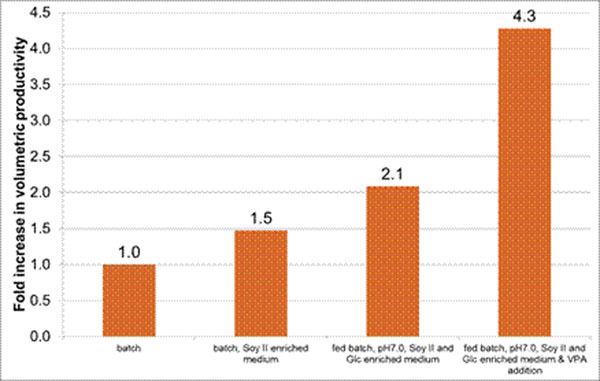
Process development C1-inhibitor expressing CAP cell line – Improvement of volumetric productivity.

The optimized fed batch strategy starting with 2g*L^-1^ extra glucose and 4g*L^-1^ soy peptone II and addition of 4mmol*L^-1^ VPA at a viable cell denstity of 3.5*10^6^mL^-1^ yields 4.3-fold higher volumetric productivity as compared to the batch culture (Fig.1). With the optimized fed batch we are able to produce 200-250 mg*L^-1^ C1-inhibitor.

